# Revisiting the Posttherapeutic Cure Criterion in Chagas Disease: Time for New Methods, More Questions, Doubts, and Polemics or Time to Change Old Concepts?

**DOI:** 10.1155/2015/652985

**Published:** 2015-10-25

**Authors:** Marta de Lana, Olindo Assis Martins-Filho

**Affiliations:** ^1^Departamento de Análises Clínicas, Escola de Farmácia, Universidade Federal de Ouro Preto (UFOP), Programas de Pós-Graduação em Ciências Biológicas (Núcleo de Pesquisas em Ciências Biológicas (NUPEB)) e Ciências Farmacêuticas (CiPHARMA), Escola de Farmácia, UFOP, 35400-000 Ouro Preto, MG, Brazil; ^2^Laboratório de Biomarcadores de Diagnóstico e Monitoração, Centro de Pesquisas René Rachou (CPqRR), Fiocruz, Belo Horizonte, MG, Brazil

## Abstract

One of the most relevant issues beyond the effectiveness of etiological treatment of Chagas disease is the lack of consensual/feasible tools to identify and certify the definitive parasitological cure. Several methods of distinct natures (parasitological, serological, and molecular) have been continuously proposed and novel perspectives are currently under investigation. Although the simultaneous use of distinct tests may offer better contributions and advances, it also leads to controversies of interpretation, with lack of mutual consent of cure criterion amongst researchers and physicians. In fact, when distinct host compartments (blood/tissues) are evaluated and explored, novel questions may arise due to the nature and sensitivity limit of each test. This short analytical review intends to present a chronological and critical overview and discuss the state-of-the-art distinct devices available for posttherapeutic cure assessment in Chagas disease, their contributions, meanings, and interpretation, aiming to point out the major gaps and propose novel insight for future perspectives of posttherapeutic management of Chagas disease patients.

## 1. Introduction

Chagas disease, caused by the parasitic protozoa* Trypanosoma cruzi*, is naturally transmitted by triatomine vectors and is endemic in 21 Latin American [1] countries. Currently this disease is present in several other countries of distinct continents (USA, Europe, Asia, and Australia) due to human migration phenomena [2] where its transmission occurs independently of the vectors by other mechanisms such as congenital, blood transfusion, transplants, and common use of syringes. About 6-7 million subjects are estimated to be infected worldwide, mostly in Latin America [1]. After the acute phase, approximately 30% of chronically infected patients will develop cardiac alterations, 10% the digestive alterations (mainly megaesophagus and megacolon), or the mixed clinical form of the disease (association of cardiac and digestive manifestations) [3] which may require complex clinical management and specific treatment. The etiological treatment of Chagas disease is currently based on the use of nifurtimox or benznidazole, developed in the 1960s and 1970s, respectively. Despite their relative proven efficacy to treat hosts during the acute phase of the disease, their effectiveness is very low when administered during the chronic phase of the disease [4]. Moreover, the therapeutic outcome also depends on intrinsic features of distinct* Trypanosoma cruzi* natural resistance [5] or genotypes, even during acute infection [6]. One of the most difficult challenges in Chagas disease treatment is the establishment of a feasible and consensual parasitological cure criterion. In general, the posttherapeutic management of Chagas disease patients involves the use of several methods of distinct natures, focused on the identification of live parasites, host immune-mediated biomarkers (specific antibodies and cellular mediated immunological features), or parasite-derived nucleic acid and/or antigens [4, 7]. Distinct serological, parasitological, and molecular tools have been continuously developed over time, leading to subsequent changes on the proposed cure criteria. Despite their incontestable contributions to point out the inefficiency of the current available drugs and clarify several aspects relevant to guide novel rational-guided drug discovery, the use of new approaches has also elicited several doubts and raised controversial ideas amongst researchers and/or physicians. One of the most relevant matters or limitations regarding the establishment of a mutual consent cure criteria relies on the long time required for complete seroreversion of the conventional serology, one of the front-line criteria included in most posttreatment cure management protocol and still considered a relevant biomarker to be adopted as indicator of* T. cruzi* infection cure by several groups in the scientific/medical community. Several studies in humans have demonstrated that the time required for the complete reversion of the conventional serology is directly proportional to the time of patient's infection. It has been expected that seroreversion may take 1–12 months for congenital infections, 1–5 years in children with less than five years of infection, 5–10 years for recent chronic infection (i.e., 12–14 years of infection), and 10–25 years or even more for late chronic infections (i.e., >15 years of infection) [4, 8]. These particularities are, besides the small number of drugs available for Chagas disease treatment and those aspects inherent to genotypic-specific drug resistance, one of the most relevant reasons that discourage physicians from prescribing therapeutic interventions and that also demotivate the patient adherence to treatment.

The purpose of this review is to present the over-time development of novel methods for cure management of Chagas disease patients and also analyze and critically discusses the interpretation of the results obtained with the use of several methodological tolls. In this context we point out the gaps, doubts, and controversial issues and propose novel insight for future perspectives for cure criteria feasible to be applied in follow-up management of treated Chagas disease patients.

## 2. Laboratorial Methods Available for Posttherapeutic Cure Assessment and Their Meaning

Table 1 summarizes the most relevant proposed methods reported for cure monitoring after etiological treatment of Chagas disease. Two categories of tests, including immunological (conventional and nonconventional serology) and parasitological methods, have been reported with applicability for cure assessment in Chagas disease treated hosts.

### 2.1. Immunological Methods (Conventional and Nonconventional Serological Approaches)

The conventional immunoserological tests, routinely applied for Chagas disease serology in clinical laboratories, comprise those methods based on the detection of IgG anti-*T. cruzi* epimastigote antigens by several techniques, including the complement fixation reaction (now in disuse), indirect hemagglutination assay (IHA), indirect Immunofluorescence test (IIF), and enzyme-linked immunosorbent assay (ELISA). The nonconventional and/or alternative methods, usually applied in reference or research laboratories, include those tests based on the detection of antitrypomastigote, amastigote, or purified/recombinant antigens by several technical fundaments, such as recELISA, immunoblotting, flow cytometry, and immunosensors.

There is a consensus that, after etiological therapeutic intervention in Chagas disease, seronegative results on conventional methods can be considered as cure criterion. However, the seropositivity on these methods is crudely interpreted as indicative of therapeutic failure in Chagas disease.

The first and classical definition of posttherapeutic cure in Chagas disease proposed [8, 9] define as “cured” those treated patients that presented negative conventional serology isolated or associated with negative parasitological tests (xenodiagnosis and/or hemoculture or other tests). Additionally, this “classical cure criterion” defines as “therapeutic failure” the presence of seropositive serology regardless of the results observed in the parasitological methods even when consistently negative (Table 1).

However, since 1982, [10] it has been reported that* T. cruzi*-infected hosts may present dissociation between the results observed in the conventional and nonconventional serology. These authors have proposed that during* T. cruzi* infection the hosts produce two distinct categories of immunoglobulins referred to as “conventional” and “nonconventional” antibodies. The first category, named “conventional antibodies,” was useful for diagnosis purposes but may persist after parasitological cure. The second category represents a special type of antibodies, named “nonconventional” antibodies or “lytic antibodies,” able to bind live* T. cruzi* trypomastigote forms leading to their complement mediated lysis. These authors observed that this type of “lytic” antibodies are closely associated with the presence of active* T. cruzi* infection but are absent in parasitological cured hosts. Considering these concepts, [10] these authors proposed the inclusion of the complement mediated lysis technique (C^o^ML) in the context of cure criteria. The C^o^ML offered an important contribution for posttherapeutic monitoring of Chagas disease, since it was able to detect early seroreversion prior to the conventional serological methods. The C^o^ML has gained strength for cure assessment of human Chagas disease after an important posttherapeutic follow-up study [11]. According to this proposal, a new concept of cure arises by the introduction of the term “dissociated” patients. By this “novel cure criterion,” treated patients presenting with positive conventional serology but negative C^o^ML should be considered cured (Table 1). The use of the [10] criterion enhances the therapeutic effectiveness from around 9% obtained by the “classic cure criterion” to approximately 37% when including the “dissociated” patients (28%) as cured [11].

Considering the functional nature of the C^o^ML, the requirement of a fresh complement source (animal or human serum) and the use of live blood trypomastigote (obtained from irradiated mice or tissue cultures) besides the laborious counting system in Neubauer chamber and the low sensibility (using sera dilution 1 : 2 to 1 : 8), this method found restriction for general use and acceptance by the scientific community. In 1995, aiming to overcome some limitations inherent of C^o^ML, a novel approach for detection of anti-live trypomastigotes antibodies, analogous to “lytic” antibodies, was proposed, based on the use of flow cytometry. This new method, referred to as flow cytometry anti-live trypomastigote antibody (FC-ALTA), was first described [12] and performed in parallel with C^o^ML. This method overcomes the functional nature of the C^o^ML, substituting the fresh complement by FITC-conjugated anti-human IgG, in a flow cytometry-based immunofluorescence approach. This innovation contributes to the development of a more sensitive (serum dilution 1 : 256) and reproducible flow cytometric-reading reaction. The FC-ALTA performance led to the same patient categorization obtained by the C^o^ML, distinguishing “not-treated” (NT) and “treated-not-cured” (TNC), with positive results, from those “dissociated” (DIS) or “cured” patients (TC), with negative results. Later on, the FC-ALTA was validated on a double-blind study, using a larger number of samples [13].

Aiming to overcome the biohazard of using live trypomastigote, a new flow-cytometry methodology using pre-fixed* T. cruzi* epimastigotes forms was described, easily obtained in large scale by axenic* in vitro* culture in LIT medium [14]. This novel approach was named flow cytometry detection of anti-fixed epimastigote antibodies (FC-AFEA). Both FC-ALTA and FC-AFEA methods are revealed to be able to discriminate “cured” from “not-treated” (NT) and “treated-not-cured” (TNC) patients in a long-term (more than 20 years) follow-up study. However, it has been reported [15] that while FC-ALTA is able to detect early changes in short-term posttherapeutic follow-up (5 years), no changes in the FC-AFEA could be observed.

More recently, a remarkable innovation has been incorporated in the flow-cytometry-based methodology by the introduction of a triplex concept that simultaneously uses the three* T. cruzi* evolutive stage forms amastigote (A) and trypomastigotes (T) from tissue cultures and epimastigotes (E) from axenic LIT culture [16]. This novel methodology, named FC-ATE, applies fluorescent FITC-based discrimination of* T. cruzi* evolutive forms, coupled by a fluorescent phycoerythrin-based development system to detect* T. cruzi* stage-specific antibodies. This method was able to detect “cured” patients with outstanding performance (100%) for anti-amastigote antibodies as compared with anti-trypomastigotes (93%) or anti-epimastigotes (96%) antibodies.

In consonance with the development of nonconventional serological approaches, several novel antigenic preparations, including semipurified/purified/recombinant antigens as well as synthetic peptides, have been used with the purpose of early detection of seroreversion after etiological treatment of Chagas disease [17–19], most of them using ELISA-based tests.

Also, the recELISA using specific* T. cruzi* antigens (cytoplasmic repetitive antigen (CRA) and flagellar repetitive antigen (FRA)) has shown, besides good correlation with the conventional serology (CS) and the ability to anticipate the seroreversion in some patients [20]. Moreover, the use of ELISA-F29 test has revealed negative results within patient with negative or declining conventional serology suggesting that the ELISA-F29 test is useful as an early indicator of negative seroreversion in treated chronic patients [21].

### 2.2. Parasitological Methods

The parasitological methodologies applied to cure control of Chagas disease include two categories referred to as (1) direct parasitological tests, including fresh-blood-examination (FBE) and trypomastigotes concentration (buffy coat, microhematocrit, and Strout tests) and (2) indirect parasitological tests, including xenodiagnosis and hemoculture [4].

Despite its low sensitivities, a positive parasitological test is considered as definitive evidence of therapeutic failure, even isolated or in association with immunological test since it detects the presence of live parasites in host peripheral blood [4, 8]. On the other hand, a negative parasitological result does not confirm the therapeutic effectiveness or discard the possibility of therapeutic failure. Usually, the direct parasitological tests are useful for cure monitoring of treatment performed during acute and subacute phases.

In general, all parasitological methodologies are employed in parallel with serological tests in order to provide a more precise cure criteria definition. In these cases, a negative parasitological test in consonance with a patent seroreversion phenomenon is considered the final proof of therapeutic success.

### 2.3. Molecular Methods (PCR and qPCR)

In the 1990s, the use of molecular methods has been proposed, mainly to overcome the low sensitivity of most parasitological approaches. In this context, the polymerase chain reaction (PCR) appeared having as target of amplification the variable region of the minicircles of the kDNA [22]. Taking into account its high sensibility, the PCR was immediately used in the context of cure control, shown to be the first to demonstrate the therapeutic failure on a short-term basis and the last to become negative in cases of therapeutic success, as compared to all parasitological methods employed for cure control in humans [23–25] and experimental models treated by the conventional or alternative therapeutic schemes [26, 27]. For this reason, the PCR has become a useful tool for therapeutic failure definition amongst all parasitological cure control methods [23, 28] since the detection of the* T. cruzi* k-DNA in peripheral blood samples eluate was interpreted as indicative of the parasite presence in the host (Table 2). However, later on, several reports have demonstrated that, despite the higher sensitivity of PCR as compared to other parasitological methods, the PCR still presents some sensitivity limitation to detection of parasite DNA in chronic infections, as well as in treated hosts [26, 29]. Therefore, it became mandatory that a negative PCR cannot be interpreted alone as indicative of parasitological cure and also requires, likewise other parasitological test, a parallel seroreversion evidence for conclusive cure report.

Later on, the PCR-based methodologies have evolved towards quantitative methods using fluorescent-based probes q-PCR that represent advantages over the conventional PCR, being more sensitive and able to estimate the parasitemia levels in a quantitative manner which is relevant to monitor patients and host before and after etiological treatment. Two main molecular targets have been explored in the amplification of the parasite DNA by qPCR: the minicircle k-DNA [30] and the satellite DNA [31]. The higher sensitivity of qPCR besides the possibility of estimating the parasitemia levels has been explored as the major immediate and/or permanent advantages of this method in clinical and experimental studies. The good correlation between the qPCR data with the blood parasitemia and intensity of tissue lesions as well as electrocardiographic alterations have been already reported [32]. Moreover, the applicability of qPCR to monitor patients [33] and animal models [34] after etiological treatment with conventional or alternative new drugs has been also addressed.

It is important to mention that both PCR and qPCR can be used in the analysis of peripheral blood samples and also in several host tissues (biopsy or euthanized experimental animals) and therefore represent an important contribution in the parasitological cure monitoring of animals and humans hosts [34, 35].

### 2.4. New Insights on Cellular Immune Response Methods

Aiming to improve novel concepts and bring novel insights to the therapeutic efficacy monitoring approaches in Chagas disease, new studies evaluating the* T. cruzi-*specific cellular immune response have been published, trying to surrogate the “old concept” that only seroreversion should be considered as the absolute indicative of cure. In this context, it has been proposed that changes in specific anti-*T. cruzi* T-cell responses, mainly focusing on the IFN-*γ* production by NK-cells and CD8^+^ memory T-cells and/or IL-10 produced by B-cell and monocytes should be explored [36, 37]. Some reviews about this theme suggest that despite the difficulty to monitor cellular immunity biomarkers, due to their low frequency and broad antigen specificity, the tracking of theses target biomarkers by phenotypic analysis of* T. cruzi*-specific immune response can represent a promising tool for posttherapeutic evaluation in Chagas diseases hosts. There is a general consensus that, regardless of the fact that parasitological cure cannot be achieved in some cases, the ability of the etiological treatment to shift the immune response towards a mixed and balanced profile, with a IL-10-modulated and IFN-*γ*-driven-proinflammatory microenvironment, is a benefit to be considered even in not cured patients. Several immunological mediators have also been explored with this purpose [36–38].

## 3. Discussion, Gaps/Doubts, and Conclusions

The first experimental studies of etiological treatment in Chagas disease were carried out in mice in 1961 [39] and the posttherapeutic evaluation was performed by fresh blood examination (FBE) followed by serological test [39]. However, the first report of nitrofuran compounds use for treatment of human patients with chronic Chagas disease was published in 1969 [40] with posttreatment evaluation performed by xenodiagnosis and conventional serology. The first clinical trial of posttherapeutic evaluation following etiological treatment in human Chagas disease has used the xenodiagnosis and hemoculture, both of low sensitivity, but with an important advantage to clearly demonstrate the therapeutic failure in cases of positive results [4]. Although the xenodiagnosis has been proposed for use in experimental studies and also in humans, the ethical limitations and current controversies regarding this technique must be considered.

The conventional serological tests (CFR, IIF, IHA, and ELISA) for detection of specific antibodies against* T. cruzi* were usually used in parallel with parasitological methods, especially due to their high sensitivity and relative high specificity. The first “classic cure criterion” proposed for posttherapeutic monitoring of humans Chagas disease and* T. cruzi* experimental infection was based on the use of combined parasitological/conventional serology approaches and suggested that treated hosts should be considered cured only when all tests show negative results, indicating parasitological clearance and seroreversion [8, 9]. By using this “classic cure criterion,” the initial studies on postchemotherapy follow-up in humans reported the time required for the seroreversion of conventional serology were very long, even when the parasitological methods were persistently negative, leading to resilient dissatisfaction of the scientific community and especially physicians working with human or experimental etiological treatment [41]. Considering the “classic cure criterion” based on the conventional serology (CFR, IHA, IIF, and ELISA) and considering “cure” only the cases of patent seroreversion, most studies revealed that, despite the fact that the therapeutic success during acute/subacute Chagas disease could reach over 90%, the frequency of “cured patients” drops significantly to around 9% when the treatment was performed during chronic infection [8, 9]. It has been proposed that a rapid and intense decrease in conventional serological titers after etiological treatment could be considered an indicative of putative cure [42].

A promising proposal to overcome the long time required for cure assessment by the conventional serology methodologies became available with the detection of anti-live trypomastigote antibodies, initially by the C^o^ML [10, 11] and later on by flow cytometry (FC-ALTA, FC-AFEA, and FC-ATE) [12, 14–16]. By using these nonconventional serological approaches, a novel “Krettli and Brener cure criterion” has been proposed [10], offering the important advantages, revealing more rapid seroreversion or demonstrating decreasing serological titers of antilive trypomastigotes, even when the conventional serology remained unaltered after treatment. The detection of these special antibody types appears to be associated with the presence of active infection in hosts, both humans and experimental animals [24, 27]. It has been demonstrated that FC-ALTA was negative in the majority of cured patients and in some patients with positive conventional serology [25], indicating the presence of “dissociated” patients, according to the “Krettli & Brener cure criterion” [10]. Despite the contribution of the nonconventional serological approaches anticipating the therapeutic efficacy outcome, these methods have not been effectively introduced in the routine of clinical laboratories for posttreatment evaluation, probably due to technical particularities and methodological difficulties. It is important to mention that the use of semiquantitative serology by flow cytometry is possible to detect decreasing serological titers, even when the anticipation of seroreversion is not yet observed in humans [15, 43] and in isolated cases in murine model [44]. It is important to mention that the standardization, development, and availability of a single, simple, inexpensive, easy to handle, sensitive, and specific method for cure criterion assessment are absolutely desirable. From this point of view, the FC-ALTA is useful to establish differences between conventional and nonconventional antibodies following Chagas disease chemotherapy. However, this technique still remains restricted to reference laboratories, especially due to the use of live trypomastigotes* T. cruzi* cultures, the requirement of a flow cytometer and trained personnel for standardization, and interpretation of the results.

It is possible that* T. cruzi* genetic variability present in infected hosts could be involved in the time-dependent seroreversion observed in some hosts. Genotypic-specific serology using antigens (whole parasites, purified proteins, or peptides) from distinct* T. cruzi* genetic groups to identify strain-specific infections in humans and experimental models is currently in progress by several teams in order to search and achieve higher specificity in the serological tests. Probably, these methods will be available in a near future for cure assessment in genotype-specific approaches.

It is unquestionable that the use of the “Krettli and Brener cure criterion” [10] enhanced the therapeutic effectiveness from around 9% to approximately 37% when including the “dissociated” patients as cured [11]. Although the acceptation of the “dissociated patients” as parasitological cured patients is still questioned, mainly by the physicians, this concept has increased progressively after the introduction of more sensitive parasitological methodologies in the context of posttherapeutic monitoring of patients. It has been frequently observed that negative PCR results can be associated with negative nonconventional serology even in hosts with positive conventional serology, reenforcing the existence of this category of cured hosts with dissociation between positive conventional serology and negative nonconventional serology.

The introduction of PCR-based methodologies with higher sensitivity, to overcome the gaps of traditional parasitological approaches (xenodiagnosis and hemoculture), offered important contributions in the context of Chagas disease cure control and still supports an improved identification of the parasite persistence after etiological treatment. The questions around whether positive results observed in PCR-based methods in treated Chagas disease patients are really due to the presence of live parasite still remain to be elucidated. Successive follow-up evaluations in humans must be performed in order to better understand the real meaning of positive PCR in the context of cure monitoring, in parallel with other methodologies indicated for posttherapeutic evaluations. However, it is important to mention that, in murine model [45], positive results in PCR of blood eluates can be considered a “definitive indication” for the presence of live parasites, since only intact or recently lysed parasites are able to yield persistent positive results on these methods [46]. Otherwise, PCR of blood eluates may have a short-term positivity, up to two days, when mice are inoculated with purified* T. cruzi* DNA [46]. Therefore, the positivity in PCR-based methods assumed the “gold standard” score for therapeutic failure assessment [46]. On the other hand, there is general consensus that the combination of negative results of PCR-based methods in consonance with negative conventional serology approaches is definitive indication of posttherapeutic cure in Chagas disease hosts [23, 33, 46]. Important contributions have been also observed in the posttherapeutic evaluation in experimental models (mice and dogs) showing good correlation between negative PCR and negative parasitological tests (xenodiagnosis and hemoculture), conventional serology, and nonconventional serology as consensus for cure assessment [26], likewise observed in humans [23]. It is important to mention that although the qPCR has been identified as promising method, this approach, likewise conventional PCR, detects DNA from live and also dead/disintegrated parasites. Moreover, the use of qPCR still remains restricted to specialized laboratories and research centers.

All studies using PCR-based methods for follow-up monitoring of Chagas disease treated patients have pointed out that, likewise the phenomenon observed in the serological approaches, there is a progressive change in the result profiles, starting with oscillating results, interpreted as fluctuant parasitemia, before definitive negative results [23, 25, 33]. However, the time required for negativation of PCR-based methods is not clear yet. After several years, or even decades, of posttherapeutic follow-up by distinct methodologies, several studies have revealed important contradictions amongst PCR and serological results (conventional and nonconventional). It has been demonstrated that when PCR and/or nonconventional serology were negative, oscillating titers in the conventional serology may indicate a tendency of putative cure [44]. These evidences have been shown in a ten-year posttherapeutic follow-up in children and also with experimental infections with the same* T. cruzi* strains [44]. These findings suggested that PCR, likewise nonconventional serology, may take lower time for negativation as compared to conventional serology. In agreement with this proposal, the publications [21, 44] verified that the cure may be confirmed in patients with lowering serological titers after treatment when they are evaluated by ELISA using another antigen.

It has been demonstrated that the targets of the host-immune response mediated by conventional and nonconventional anti-*T. cruzi* antibodies are distinct [47]. While the nonconventional antibodies require the presence of live parasites since they are direct to short-lasting GP160 membrane-bound antigens, the conventional serology antibodies recognize a large range of antigens and can become positive even after mice immunization with soluble* T. cruzi* antigens or dead parasites. Several reasons may explain the long time required for the complete reversion of conventional serology, including mechanisms of autoimmunity.

Moreover, the persistence of parasite antigens in the dendritic and cardiac cells [47, 48], the occurrence of anti-idiotypic antibodies mimicrying parasite epitopes, and the presence of anti-laminin and anti-carbohydrate epitopes antibodies as well as cross-reactivity with other microorganisms such as intestinal or lung bacterias/protozoa may count for the long-term persistence of residual conventional serology after effective treatment of Chagas disease host [47]. Taking into account all these factors, an important question may arise: “*is it really necessary to consider the negativation of the conventional serology as the only way for discrimination between cured and not cured patients?”*

The current knowledge about the different performance of methods comparatively applied for posttherapeutic evaluation of* T. cruzi* hosts (humans and experimental models) leads the group of interdisciplinary experts in Chagas disease chemotherapy [49] to purpose a new protocol for cure monitoring for the evaluation of new compounds in mouse model. Considering the long time required for negativation of conventional serology and the high sensitivity of PCR applicable for cure control, the conventional serological tests have been ruled out of the most recent cure criteria that recommend the use of PCR after immunosuppression, performed 30 days after the end of the etiological treatment, as the “reference standard criteria” for cure assessment.

The introduction of qPCR approaches, with additional gain in quantitative analysis of parasitemia associated with high sensitivity level, has offered additional advantages over the original PCR-based methods applied for posttherapeutic evaluation in Chagas disease. The expectation regarding qPCR-based methods is naturally very high, regardless of the few data already available, based on follow-up studies in human. In general, in experimental models the use of qPCR has appeared to have a direct correlation between the level of parasitemia and the intensity of tissue parasitism, inflammatory process, and level of proinflammatory chemokines and fibrosis [34]. Even with all these optimistic features, the same old questions raised to interpret the PCR results remain for qPCR: “*is a positive qPCR result related to the presence of live parasites or dead/disintegrated parasites could also lead to positive results?”* Moreover, it is important to remark that, even considering all the advantages aggregated by PCR and qPCR methodologies, they still have some limit of sensitivity, especially when intended to detect extremely low parasitemia levels like that observed in treated chronic patients. Moreover, as the PCR-based methods are not available yet in most regular primary health care units, even in endemic regions of endemic countries, only physicians that collaborate with research institutions have employed these methods in the posttherapeutic management of Chagas disease patients.

Another question regarding the use of PCR-based methods has been raised by comparative performance of these methods to yield positive results depending on the biological samples used. Surprising and intriguing results have been demonstrated when PCR-based methods are applied to distinct tissues and compared to blood eluate in experimental models for* T. cruzi* infection chemotherapy. The first report for inconsistent positive tissue PCR in mice considered cured by negative conventional serology, hemoculture, and PCR was documented [50]. Afterwards, [27] a more worrying situation of inconsistence in positive tissue PCR even in treated mice presenting negative nonconventional serology (FC-ALTA) was showed. Similar inconsistences were observed between blood and tissue when qPCR methods are applied immediately after the end of the therapeutic intervention, suggesting that, in some cases, the qPCR negativation in blood samples happens earlier than in the heart tissue [34]. Together, these findings may suggest that the negativation of PCR in the blood samples may occur before the parasite clearance in the tissues. Another possibility is that the parasite DNA detected in the tissues may derive from dead or disintegrated parasite and is not indicative of the presence of live parasites in the tissue. In this context, the methods based on immunosuppression have been suggested to be used for cure monitoring assessment in experimental models. This experimental approach may contribute to elucidate these queries. One alternative that has also been proposed for use in experimental protocols during drug development research is the use of immunohistochemistry methodologies in tissue [51] for direct detection of amastigotes or* T. cruzi* antigens in tissue samples in parallel with PCR or qPCR. However, the relative low sensitivity of immunohistochemistry for parasite detection represents important restrictions for its use in humans.

Taken together, in the scenario of all these tolls available for the posttherapeutic monitoring of* T. cruzi* infection in human and experimental models, there is a clear and natural theoretical hypothesis for a decreasing order of negativation as follows: xenodiagnosis/hemoculture > PCR (blood eluate > tissues) > qPCR (blood eluate > tissues) > nonconventional serology > conventional serology.

The goal to be achieved is to disseminate this novel concept of posttherapeutic cure in Chagas disease and to stimulate physicians to pursue these laboratorial methods together in the posttreatment evaluations to undertake a more holistic view for the large benefits underlying the etiological treatment of this disease. To reach this goal, it is necessary to change old concepts. Unfortunately, there is not ethical possibility to determine the treatment efficacy in human* T. cruzi* infection with 100% conviction tissue [36]. It may not be mandatory to unequivocally demonstrate the absence of live parasites in host blood and tissues but to take the whole set of posttherapeutic changes in parasitological (persistent negative xenodiagnosis and/or hemoculture along with negative/oscillating PCR and/or qPCR in blood eluates) and immunological results (negative/decreasing titers in nonconventional and/or conventional serology besides the presence of modulated proinflammatory patterns).

## 4. What Is Still Necessary to Be Accomplished regarding the Posttreatment Evaluation?

In fact, the extension of human studies during longer period of posttherapeutic follow-up to characterize the behavior of parasitological/molecular/immunological technologies in order to verify changes in the results is still extremely important and necessary. The ideal is that all these evaluations could be taken together to achieve a pattern of biological signatures including parasitological and immunological parameters in parallel, taking the novel concept of systems biology and machine learning tools. It may not be a time to pursue new methodologies but a time to revisit the old or ancient methods for better interpretation of theirs results in the context of integrated parasitological/immunological posttherapeutic management. Meanwhile, there are increasing evidences that the etiological treatment bring benefits to the Chagas disease patients, even for the “classical not cured patients,” which evolve with better prognosis.

In this meantime, equivalent approaches of posttherapeutic evaluation should be conducted in experimental models, focusing on novel tools and interpretation approaches, with the advantages that the results can be generated more promptly in a more controlled, precise, and repetitive investigation, besides the possibility of parallel tissue investigation, including tissue biopsy culture to verify the presence of live parasites. Another plausible tool that has been recently applied in possibility in animals is the use of posttreatment immunosuppression to monitor the therapeutic efficacy, in order to verify the presence of live parasites in tissue [49, 52].

## 5. What Is Desirable?

The standardization, development, and availability of a single, simple, inexpensive, easy to handle, sensitive, and specific method for effective demonstration of the parasite presence in blood (presence of the parasite DNA and/or antigens in blood samples) are absolutely desirable. While this task appears difficult to be accomplished, it is time to change old concepts and accept novel models to look at old or ancient methods in the context of integrated multidisciplinary posttherapeutic management. In the future, an integral concept should include clinical evolution of treated patient, especially in long-term follow-up studies. The posttherapeutic Chagas disease monitoring should not be restricted to laboratorial analysis. It should be extended to an integrated clinical/laboratorial status of treated patients.

It is clear that a first and critical step to address the research and development gap regarding the establishment of an international approved “cure criterion” is to establish consensus on the desirable target product profiles (TPPs) in different conditions of use. To support this process and optimize the development of such tools, the Pan American Health Organization (PAHO), in collaboration with the Drugs for Neglected Diseases initiative (DNDi), Médecins sans Frontières (MSF), and The Special Program for Research and Training in Tropical Diseases (TDR), convened a multidisciplinary group of experts to review the “state of the art” regarding this matter and initiate discussions. The multidisciplinary group has prepared for the development of TPPs, which has been recently reported [53].

## 6. Conclusions

The etiological treatment of human Chagas disease acts to reduce or eliminate the etiological agent* T. cruzi*, changing the patterns of anti-*T. cruzi* antibodies and remodeling the antiparasitic cellular immune response. Several laboratorial tests of parasitological or immunological nature are available and are usually employed for posttherapeutic cure monitoring. The major dissatisfaction affecting the scientific community and physicians relies on the long time required for persistent seroreversion (10–25 years or even more), which has been classically considered a definitive parasitological cure criterion. Although a range of novel methodologies of distinct and more sophisticated theoretical basis (parasitological, serological, or novel immunological biomarkers) have appeared and applied in the context of posttherapeutic monitoring of Chagas disease, this theme still remains polemical due to controversies between results obtained by distinct tests and/or in different studies. Always when a new test is proposed to be used as a tool for cure monitoring, it faces the lack of a plausible short-term “gold standard” methodology that could be applied to validate the proposed method. Moreover, all initiatives for new or improved tools for cure control must overcome the hard challenge to be sensitive and specific enough to demonstrate the clearance of the live parasite in host tissues. In this context, it seems that more than new methods about what is needed is an urgent change of old concepts. It is time to revisit the methods available and propose better interpretation of their results in the light of an integrated system biology posttherapeutic management, intending to establish a holistic view of the* T. cruzi* hosts as a complex network of biomarkers that could be better assimilated and understood by a multiparametric prognostic equation.

## Figures and Tables

**Table 1:** Laboratorial methods used for posttherapeutic cure criteria in human Chagas disease and *Trypanosoma cruzi* infection in experimental models^*∗*^.

Conventional serological tests	Nonconventional serological tests	Parasitological test	Interpretation
(CFR, IHA, IIF, and ELISA)	(C^o^ML, FC-ALTA, FC-AFEA, and FC-ATE)	(Direct test, indirect tests, and PCR)	
POS	POS	POS	Not cured
NEG	NEG	NEG	Cured (classic criterion)
POS	NEG	NEG	Dissociated/cured (Krettli and Brener criterion)
POS/NEG	POS/NEG	NEG	Oscillating/inconclusive

^*∗*^Conventional serological tests: CFR (complement fixation reaction); IHA (indirect hemagglutination assay); IIF (indirect Immunofluorescence test), and ELISA (enzyme-linked immunosorbent assay); nonconventional serological tests: C^o^ML (complement mediated lysis); FC-ALTA (flow cytometry anti-live trypomastigotes antibodies); FC-AFEA (flow cytometry anti-fixed epimastigotes antibodies); FC-ATE (flow cytometry anti-live amastigote/trypomastigotes and fixed epimastigote antibodies); direct parasitological tests: fresh blood examination (FBE), trypomastigotes concentration (buffy coat, microhematocrit, and Strout tests); indirect parasitological tests: xenodiagnosis, hemoculture, and PCR.

**Table 2:** Categories of laboratorial methods and targets available for evaluation of the etiological treatment efficacy/benefits and their interpretation in the context of Chagas disease cure assessment^*∗*^.

Category	Laboratorial method	Target	Results	Interpretation
Conventional serological tests	CFR, IHA, IIF, and ELISA	Fixed epimastigotes Recombinant antigens	POS	Therapeutic failure (Classic criterion)
			NEG	Cured (Both criteria)
Nonconventional serological tests	C^o^ML FC-ALTA, FC-AFEA, and FC-ATE	Live trypomastigotes Live amastigotes	POS	Therapeutic failure (Krettli and Brener criterion)
			NEG	Cured (Krettli and Brener criterion)
Direct parasitological tests	FBE, trypomastigote concentration (buffy coat, microhematocrit, and Strout tests)	Blood trypomastigotes	POS	Therapeutic failure (Both criteria)
			NEG	Inconclusive (Both criteria)
Indirect parasitological tests	Xenodiagnosis, hemoculture	Blood trypomastigotes	POS	Therapeutic failure (Both criteria)
			NEG	Inconclusive (Both criteria)
Indirect parasitological tests	PCR, qPCR	Parasite DNA	POS	Therapeutic failure (Both criteria)
			NEG	Inconclusive (Both criteria)
Cellular immunology test^#^	PBMC/whole blood cultures/flow cytometry	*T. cruzi*-specific INF-*γ*/IL-10 producing cells	Proinflammatory	Side effect
			Balanced profile	Benefit

^*∗*^Conventional serological tests: CFR (complement fixation reaction), IHA (indirect hemagglutination assay), IIF (indirect immunofluorescence test), and ELISA (enzyme-linked immunosorbent assay); nonconventional serological tests: C^o^ML (complement mediated lysis), FC-ALTA (flow cytometry anti-live trypomastigotes antibodies), FC-AFEA (flow cytometry anti-fixed epimastigotes antibodies) and, FC-ATE (flow cytometry anti-live amastigote/trypomastigotes and fixed epimastigote antibodies); direct parasitological tests: fresh blood examination (FBE) and trypomastigotes concentration (buffy coat, microhematocrit, and Strout tests); indirect parasitological tests: xenodiagnosis, hemoculture, PCR, and qPCR; cellular immunology test: peripheral blood mononuclear cell and/or whole blood in vitro cultures in the presence of *T. cruzi*-derived antigens followed by cell surface phenotypic analysis and intracellular cytokine staining by flow cytometry. ^#^Proinflammatory pattern refers to IFN-*γ* mediated immune response of NK-cells and CD8^+^ T-cells. Balanced profile refers to IL-10 modulated response by monocytes or B-cells.
